# Prognostic Value of Deep Learning-Mediated Treatment Monitoring in Lung Cancer Patients Receiving Immunotherapy

**DOI:** 10.3389/fonc.2021.609054

**Published:** 2021-03-02

**Authors:** Stefano Trebeschi, Zuhir Bodalal, Thierry N. Boellaard, Teresa M. Tareco Bucho, Silvia G. Drago, Ieva Kurilova, Adriana M. Calin-Vainak, Andrea Delli Pizzi, Mirte Muller, Karlijn Hummelink, Koen J. Hartemink, Thi Dan Linh Nguyen-Kim, Egbert F. Smit, Hugo J. W. L. Aerts, Regina G. H. Beets-Tan

**Affiliations:** ^1^ Department of Radiology, Netherlands Cancer Institute - Antoni vanLeeuwenhoek Hospital, Amsterdam, Netherlands; ^2^ GROW School for Oncology and Developmental Biology, Maastricht, Netherlands; ^3^ Artificial Intelligence in Medicine (AIM) Program, Brigham and Women’s Hospital, Harvard Medical School, Boston, MA, United States; ^4^ Affidea, Cluj-Napoca, Romania; ^5^ Department of Neuroscience, Imaging and Clinical Sciences, Gabriele D’Annunzio University of Chieti, Chieti, Italy; ^6^ Department of Thoracic Oncology, Netherlands Cancer Institute - Antoni van Leeuwenhoek Hospital, Amsterdam, Netherlands; ^7^ Department of Pathology, Netherlands Cancer Institute - Antoni van Leeuwenhoek Hospital, Amsterdam, Netherlands; ^8^ Department of Surgery, Netherlands Cancer Institute - Antoni van Leeuwenhoek Hospital, Amsterdam, Netherlands; ^9^ Institute of Diagnostic and Interventional Radiology, University Hospital Zurich, Zürich, Switzerland; ^10^ Radiology and Nuclear Medicine, University of Maastricht, Maastricht, Netherlandsa; ^11^ CARIM School for Cardiovascular Diseases, University of Maastricht, Maastricht, Netherlands; ^12^ Department of Radiology, University of Southern Denmark, Odense, Denmark

**Keywords:** artificial intelligence, immunotherapy, checkpoint inhibitors, non small cell lung cancer, treatment monitoring

## Abstract

**Background:**

Checkpoint inhibitors provided sustained clinical benefit to metastatic lung cancer patients. Nonetheless, prognostic markers in metastatic settings are still under research. Imaging offers distinctive advantages, providing whole-body information non-invasively, while routinely available in most clinics. We hypothesized that more prognostic information can be extracted by employing artificial intelligence (AI) for treatment monitoring, superior to 2D tumor growth criteria.

**Methods:**

A cohort of 152 stage-IV non-small-cell lung cancer patients (NSCLC) (73 discovery, 79 test, 903CTs), who received nivolumab were retrospectively collected. We trained a neural network to identify morphological changes on chest CT acquired during patients’ follow-ups. A classifier was employed to link imaging features learned by the network with overall survival.

**Results:**

Our results showed significant performance in the independent test set to predict 1-year overall survival from the date of image acquisition, with an average area under the curve (AUC) of 0.69 (*p <* 0.01), up to AUC 0.75 (*p* < 0.01) in the first 3 to 5 months of treatment, and 0.67 AUC (*p* = 0.01) for durable clinical benefit (6 months progression-free survival). We found the AI-derived survival score to be independent of clinical, radiological, PDL1, and histopathological factors. Visual analysis of AI-generated prognostic heatmaps revealed relative prognostic importance of morphological nodal changes in the mediastinum, supraclavicular, and hilar regions, lung and bone metastases, as well as pleural effusions, atelectasis, and consolidations.

**Conclusions:**

Our results demonstrate that deep learning can quantify tumor- and non–tumor-related morphological changes important for prognostication on serial imaging. Further investigation should focus on the implementation of this technique beyond thoracic imaging.

## Introduction

Recent advancements in the understanding of the tumor-immune cell interactions (1, 2) have enabled the development of novel drugs for the treatment of advanced-stage lung cancer. Immune checkpoint inhibitors, in particular, have been shown to provide sustained clinical benefit to patients, especially in the metastatic setting (3–5).

Metastatic markers that can be used for patient selection (i.e., before the start of treatment), as well as for treatment monitoring (i.e., during treatment), are still under research (6–8). In the context of oncological research, most predictive/prognostic markers are derived from tissue samples, routinely-extracted blood (9), or non-invasive radiological imaging (surrogate imaging markers). Tissue samples derived from biopsies (usually taken from anatomically accessible locations) often fail to account for inter- and intra-lesion heterogeneity, and response assessed during evaluation of tissue samples of only a few lesions does not necessarily mean that all lesions have responded in the same way. Furthermore, serial biopsies during longitudinal follow-up are cumbersome for the patient but also impractical. Regardless of biomarker source, monitoring of response to therapy remains challenging. As such, they are not part of the routine clinical workflow of patients.

Standard clinical imaging provides a non-invasive overview of the entire tumor burden and has the potential to more accurately evaluate the overall response of the patient to the treatment. Yet, imaging evaluation is currently limited to 2-dimensional “subjective” measurements of tumor size changes (10), time-consuming ROI delineation (11, 12), and/or to values approximating metabolic activity (*i.e., SUV values in PET*) (6). By limiting the use of imaging for response evaluation to only these approaches, many (potentially prognostic) imaging characteristics are ignored. For example, as the disease evolves in multiple distal sites, traditional imaging assessment methods would not account for the microenvironment of each lesion, despite the fact that several potential prognostic factors (e.g., angiogenesis, inflammation, and lymphocytic infiltration) likely depend on that environment (13). Since immunotherapy is a systematic treatment modality, changes indicating response are not limited to one location but can occur all over the body. This is particularly relevant in patients treated with anti PD-1 blockade where lymphadenopathy (14, 15), parenchymal inflammations, edema (16, 17), and compression atelectasis (18), can be observed. Ideally, during image response evaluation these conditions, together with tumor growth, should be monitored and quantified as they might hold valuable prognostic information.

Using artificial intelligence (AI), treatment monitoring tools can be built, capable of rapidly assessing gross morphological changes between two (or more) follow-up images of the same patient (19), in a fully-automatic manner, completely independent of human input. In this context, image registration can be used as the basis for such a method. At its core, image-to-image registration is the process of establishing a voxel-wise match between two radiological images. By establishing a match, we can measure voxel-level differences between corresponding objects represented in the images quantitatively. While conventional registration techniques are very limited for this application, deep learning-based methods have shown promise in image-to-image registration (20). There are three main advantages to using deep learning-based image registration as the core technique. The first advantage is that registration networks are trained to match a pair of images, voxel-wise. This creates a network that is explicitly trained to quantify differences between two images. By leveraging its internal features, we can effectively obtain feature vectors that represent these voxel-wise changes. These vectors can be used for classification purposes. The second advantage of using image registration is that it can be trained on large unlabeled datasets (i.e., lacking any kind of manual annotation, such as segmentations or RECIST-like measurements), while not compromising its ability to model voxel-wise details, that are likely lost in a classical unsupervised autoencoder approach. The third advantage of using image-to-image registration is that, unlike standard RECIST, such a method could be fully automatic and not require any manual input (e.g., two-dimensional diameter measurements), and not be limited to changes in the tumor size, but it would also account for global morphological changes, whether tumor-associated or not, throughout the body. Applying an image-registration-based AI algorithm in oncological follow-up imaging enables us to develop a novel method that can accurately measure gross morphological changes during treatment. Quantitative measurements of these changes can then be used for prognostication.

This study aims to investigate the potential prognostic value of AI-mediated monitoring on CT scans in non-small cell lung cancer (NSCLC) patients receiving anti-PD-1 immune checkpoint blockade. Relying on existing technical research on image-to-image registration, we hypothesize the existence of quantitative imaging features describing a set of gross morphological changes during treatment that hold prognostic value. To test this hypothesis, we developed a deep learning network for thoracic image-to-image registration and studied the prognostic value of features learned by the network in NSCLC patients being treated with PD-1 blockade.

## Materials and Methods

### Study Cohort

For this study, we retrospectively included patients with stage IV NSCLC treated with anti-PD1 monotherapy within The Netherlands Cancer Institute-Antoni van Leeuwenhoek Hospital (NKI-AVL; Amsterdam, The Netherlands) between 2014 and 2016. All patients underwent standardized, imaging-based tumor response assessment with contrast-enhanced computed tomography (CT), with follow-up (FU) intervals of 8 to 12 weeks (**Supplement 1**). We retrieved all available FU scans within the first two years of treatment, together with a baseline scan (BL) performed 8 weeks before and up to 1 week after start of treatment. To encode pre-treatment tumor spread, a pre-baseline scan (PBL), defined as the first available scan before BL, was also retrieved when available. The exact dates of each scan were recorded with respect to the start of treatment (in days). Patients with only one scan available throughout the entire treatment regimen, or whose scan would not fully cover the thorax, were excluded from the analysis. The cohort was divided into a discovery and independent test set based on the patient identifier: patients with even ID numbers were assigned to the discovery set, patients with odd ID numbers were assigned to the independent test set. The study was carried out at the NKI-AVL with the approval of the local Institutional Review Board (IRBd19-083). This cohort is a longitudinal expansion of a previously described NSCLC cohort (11).

### Image Acquisition

The CT scans were performed by either covering the chest or covering the chest and abdomen using multi-slice CT equipment (Toshiba Aquilion CX, Minato, Tokyo, Japan; Siemens Somatom Sensation Open, Erlangen, Germany) with a tube voltage of 120 kVp, slice thickness of 1 mm, and in-plane resolution of 0.75 x 0.75 mm. The bolus injection was performed at 3 ml/s (Omnipaque 300, GE Healthcare, Chicago, Illinois, US) not pre-warmed, with a total amount based on the patient weight + 40 cc (minimum of 90 cc and maximum of 130 cc) followed by a saline flush of 30 cc. The chest CT examinations were performed 40 s after contrast injection, whereas the chest and abdomen examinations were performed at 70 s.

### Data Curation

Radiological datasets are often heterogeneous. To mitigate differences in radiological image acquisition, all CT scans were cropped between the liver and the lower neck region using the method proposed by Zhang et al. (21), and linearly resampled to 2 mm isotropic voxel size. Hounsfield units were clipped between −120 (fat) and 300 (cancellous bone) and rescaled between 0 and 1. CT scans were further cropped to 192 x 192 x 160 voxels from the center point in order to provide the network with regular image shapes during training.

### AI-mediated Quantitative Treatment Monitoring

**Figure 1:**
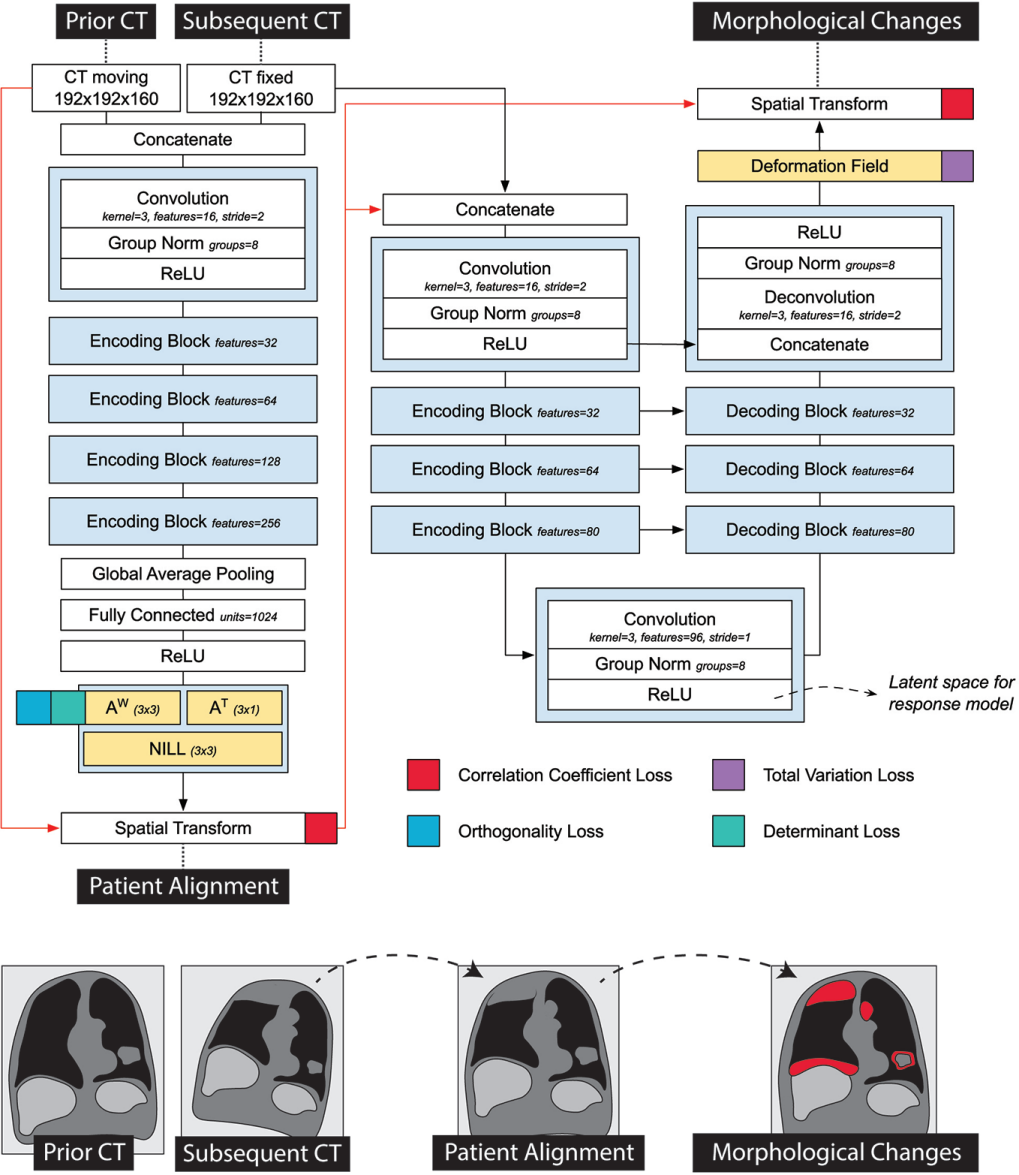
Detailed representation of the registration network used in the prognostic AI-monitoring framework.

To harness AI for quantitative treatment monitoring, we developed a 3-dimensional convolutional neural network to perform image-to-image registration between subsequent follow-ups of the same patient (architecture shown in **Figure 1**), based on the research of Balakrishnan et al. (22) and Zaho et al. (23). The network comprised of two subsequent parts: the first performing affine registration aimed to provide alignment of the scans (i.e., to correct for different patient positions), the second section performing deformable registration and aimed to identify morphological changes during the course of the treatment (i.e., longitudinal tracking).

Architecture-wise, the first part of the network consisted of a VGG-like network comprised of a series of five convolutional blocks, and two fully-connected layers, regressing the 12 parameters of the affine transform. The output transform of the network was applied to the moving image, concatenated to the fixed image, and fed into the second part of the network. The second part of the network followed a U-Net architecture (24), and it aimed to quantify non-linear anatomical differences between the input scans. This consisted of an encoding section, comprising 4 convolutional blocks downsampling the images by half the size *via* striding, a convolutional latent space with stride of 1, and four deconvolutional blocks each upsampling the inputs by double the size *via* striding. Skip connections were implemented between encoding and decoding layers following the implementation in the original paper. The network was trained to minimize the correlation coefficient loss (23). Unlike standard measurements of classical registration procedures, this loss is easy to compute in the continuous case. Three penalties were also employed to mitigate for unlikely morphological deformations, each weighted 1/10 in the final loss. Adam optimizer was used during training, with an initial learning rate of 8 × 10^−5^. A curriculum learning scheme was implemented during training, such that the loss would be computed on a smoothed version of the images. The smoothing was implemented *via* average pooling, starting with a kernel size of 9, and reduced by 3 at epochs 100, 150, and 175. Batch size was set to 2. To mitigate negative effects resulting from the small batch size, group normalization was employed instead of batch normalization. **Figure 1** shows a detailed overview of the model loss used. The network was trained on a publicly available dataset of 1010 patients of the lung image database consortium (25–27) with 10% hold out during training to control for overfitting (i.e., patients whose ID were multipliers of 10 were held out). Our code can be found online^^1^^.

### Prognostication Through Quantitative Monitoring

**Figure 2:**
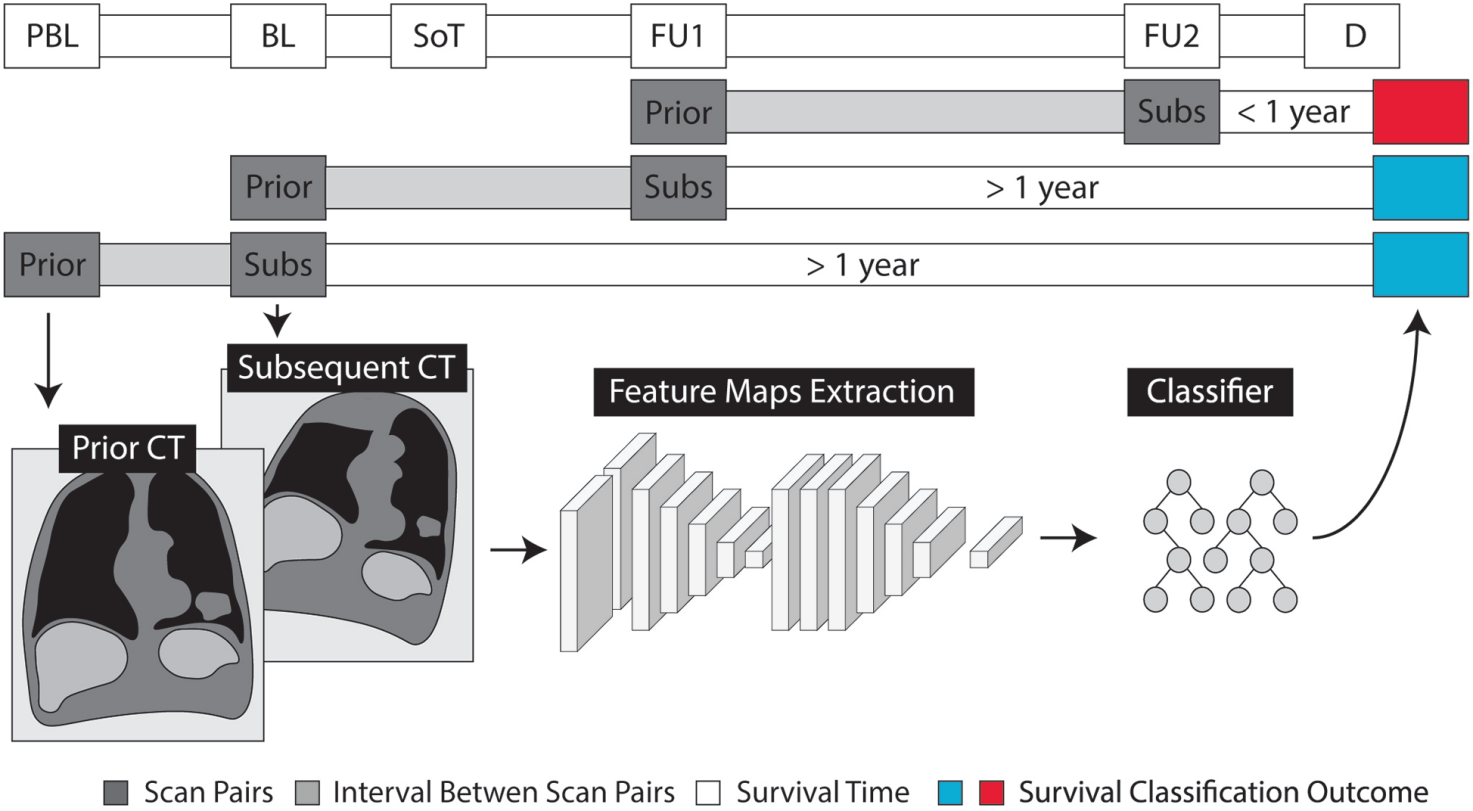
Schematic representation of the evaluation of prognostic values through quantitative monitoring. Radiological examinations are shown as pre-baseline (PBL), baseline (BL) and follow-up (FU), with respect to the start of treatment (SoT). Prediction of survival is made based on the time of death (D). For each pair of subsequent scans, we label the earlier one as *prior* and the subsequent as *subsequent* (Subs).

To explore the prognostic value of AI-mediated treatment monitoring, we trained a random forest classifier (28) (RFC), with wrapper feature selection, to predict survival based on network imaging features extracted from pairs of subsequent follow-up scans. More specifically, the RFC was trained longitudinally, on pairs of subsequent scans, to predict whether the patient would survive 1 year from the date of the latest of the two scans (see **Figure 2**). The input of the RFC consisted of 96 feature maps from the latent space of the decoder that represented the morphological changes between the prior and the subsequent scan. These are the deepest features found in the middle layer of the second section of the network—the one handling deformable registration. These features come in tensor shape, hence the name feature *maps*. For classification purposes, it is standard to transform the feature maps of the network to a feature vector, to be fed into a classifier. Global average pooling is the technique commonly used to create a feature vector out of a set of feature maps: each entry of the feature vector is the average value of the corresponding feature map. Alongside the global average pooling, we also included standard deviation, skewness and kurtosis, as we deemed the feature maps too large to be represented just by the mean activation—1,000 values per feature map, compared to 49 of a classical ResNet architecture.

To correct for temporal discrepancies (e.g., differences in time between follow-ups), the amount of days elapsed between the two scans, and the days elapsed since the start of treatment were also fed to the RFC. Furthermore, morphological changes should be order invariant: the differences estimated between image A and B should be the same as the differences between image B and A. To provide order invariance, we applied element-wise multiplication of the feature maps generated by swapping the input scans. More specifically, we computed the feature maps for the scan pair prior-to-subsequent, and the feature maps for the pair subsequent-to-prior. Then we multiplied them together, element-wise. The multiplication preserved only those changes that were detected in both directions, therefore providing order invariance to our model. The discovery set was used for training, while testing was performed on the independent validation set. Both the registration network and the random forest classifier were trained on the partitioned data, at once, with their respective default parameters—no cross-validation or model selection was performed.

### Prognostic Heatmaps

Occlusion sensitivity was employed to visualize the parts of the image that were deemed prognostic of the outcome (29). The main idea of the occlusion algorithm is based on the assumption that removing a *predictive* section/region from the original image will change the algorithm prediction substantially. In contrast, by removing a *non-predictive* section/region from the original image, the algorithm prediction will stay unchanged. We occluded a section (or patch) of the input image presented to the RFC. The prognostic value of that patch is then computed as the difference of the RFC survival score produced by the occluded image vs the original unoccluded one. The resulting prognostic map is the result of the algorithm scrolling the ROI through the image, and repeating the procedure. This was filtered with the gross morphological changes map to produce a prognostic map of the gross morphological changes used for visual interpretation. Details of the algorithm reported in **Supplement 1**. Visual assessment of the resulting prognostic maps was carried out by an expert reader (T.N.B., board-certified radiologist, 2 years experience in thoracic imaging at a tertiary oncologic center), blinded to all clinical parameters, including survival. All scan pairs were assessed with the prognostic maps overlaid on top. The reader was tasked to identify the areas of activation (i.e., hot spots) in the scan pair, and report them categorized as tumor-related areas, secondary comorbidities, and general anatomical areas. Tumor-related areas and secondary comorbidities, which were not highlighted in the prognostic map, were recorded separately.

### Independence From Known Prognostic Factors

To test the independence of our AI model, we ran a multivariate analysis against known prognostic factors. Age and pathological cancer subtypes were extracted directly from the anonymized patient records. Changes in tumoral burden were computed based on the available manual segmentations of the total tumor—i.e., all visible and segmentable lesions in the body, except for bone and brain. To ensure comparability with 2D measurements from standard RECIST criteria, volumes were converted to pseudo-diameters *via* $d=\sqrt[{3}]{}(6V/\pi )$, where V is the total tumoral burden. This computes the diameter of the sphere equi-volumetric to the total tumor burden. Tumor PD-L1 expression scoring was performed according to the instruction manual of the qualitative immunohistochemical assay developed as a complementary diagnostic tool for nivolumab (PD-L1 IHC 22C3 pharmDx, Dako, Carpinteria, CA). PD-L1 expression levels were determined by observing complete circumferential or partial linear expression (at any intensity) of PD-L1 on the plasma cell membrane of viable tumor cells. In parallel, the pattern of staining in CD4 stained slides, which also stain CD4^+^ lymphocytes and macrophages, was evaluated and compared to PD-L1 stained slides in order to avoid false positive assessment due to PD-L1 expressing macrophages in between tumor cells. Assessment of expression levels was performed in sections that included at least 100 tumor cells that could be evaluated.

### Statistical Analysis

To assess prognostic performance, the area under the receiver operating curve (ROC-AUC) was used. Confidence intervals were estimated *via* bootstrapping performed using repeated sampling with replacement (10000 times). Statistical significance was assessed *via* Mann-Whitney-U test. Kaplan Meier models were employed for survival analysis. Statistical significance of survival metrics was assessed *via* log-rank test. Prognostic (treatment monitoring) performance was quantified in terms of overall survival from the date of the scan. Biomarker performance was quantified in terms of overall survival and durable clinical benefit (complete or partial response, or stable disease, for at least 6 months) from the start of treatment. Cox-Hazards models were used for comparison of known prognostic factors.

## Results

### Study Cohort

**Figure 3:**
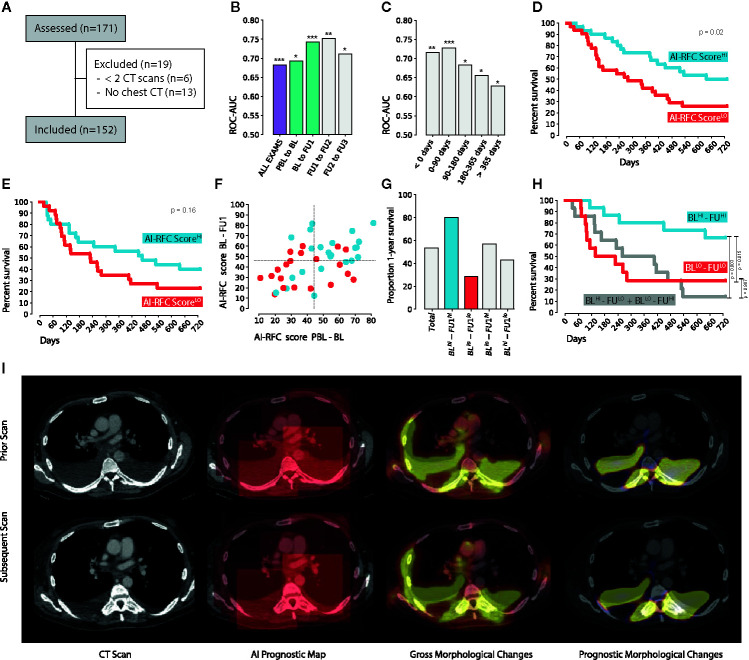
**(A)** CONSORT diagram. **(B)** 1-year survival classification performance on the independent validation set, with respect to the clinical follow-up routine (highlighted in green the ROC-AUC of the scan pairs used for the 2-years survival analysis) and **(C)** corrected by time. **(D)** 2-years Kaplan-Meier curves of the RFC survival score of BL-FU1 and **(E)** PBL-BL. **(F)** Combination of the PBL-BL and BL-FU1 RFC survival scores with **(G)** enrichment of each of the four quadrants **(F, H)** survival of each of the four quadrants. **(I)** Example of the occlusion sensitivity method used for AI explainability and visualization. * indicates p < 0.05, **p < 0.01, ***p < 0.001.

A total of 152 patients, 903 CT scans, and 611 scan matched pairs of subsequent CT scans were included in this study (see **Figure 3A**). The discovery set consisted of 73 patients (and 276 scan pairs), while the independent validation set had 79 patients (and 335 scan pairs). The median age of the entire cohort was 64.4 (IQR 57.8–68.9), with a higher prevalence of males (57.9%). Adenocarcinoma was the most common subtype, reported in 61% of the cohort. No differences in clinical characteristics were encountered between discovery and validation set, except for survival. In comparison to the discovery set, the independent validation set had 180 days longer overall survival, and 101 days longer progression-free survival. Imaging-wise, we collected 129 pre-baselines (PBL; 14.3%), 149 baselines (BL; 16.5%), 135 first follow-ups (FU1; 15.0%), and 103 second follow-ups (FU2; 11.4%). Subsequent follow-ups (FU3+) constituted the remaining 42.9% of the dataset (N = 387). Time-wise, BL scans were acquired on average 26 days before the start of treatment (IQR, 37–14), while the first FU scan, 68 days after (IQR, 46–77). Subsequent follow-ups were made on average every 77 days (IQR, 55–95). Acquisition of non-contrast-enhanced PET-CT instead of contrast-enhanced CT was the main reason for the lack of imaging during follow-up. Further patient characteristics in **Table 1**.

**Table 1:** Patient and imaging data characteristics.

	Entire Dataset	Discovery Set	Validation Set
*Patient Characteristics*			
N	152	73	79
Age [median, IQR]	64.4 (57.8–68.9)	64.5 (58.3–69.2)	64.2 (56.2–68.2)
Gender [N, %]	88 Males (57.9%)	44 Males (60.3%)	44 Males (55.7%)
Survival [median days]	341	269	449
Adenocarcinoma [N, %]	92 (60.5%)	46 (63.0%)	19 (26.0%)
Squamous [N, %]	35 (23.0%)	46 (58.2%)	16 (20.3%)
*Radiological Follow-up*			
All scan pairs	611	276	335
— PB-BL to BL [N, %]	93 (15.2%)	42 (15.2%)	51 (15.2%)
— BL to FU1 [N, %]	116 (19.0%)	55 (19.9%)	61 (18.2%)
— FU1 to FU2 [N, %]	100 (16.4%)	50 (18.1%)	50 (14.9%)
Days b/w scans in any scan pairs [median, IQR]	77.0 (55.0–95.0)	77.0 (50.0–97.2)	77.0 (56.0–94.0)
— Pre-baseline to baseline [median, IQR]	76.0 (55.0–113.0)	75.0 (47.0–114.8)	76.0 (61.0–98.0)
— Baseline to follow-up 1 [median, IQR]	85.5 (69.0–105.0)	86.0 (68.5–107.0)	85.0 (70.0–104.0)
— Follow-up 1 to follow-up 2 [median, IQR]	57.0 (44.0–78.2)	53.5 (43.0–75.0)	72.0 (47.5–83.5)
Days b/w treatment start and BL [median, IQR]	−26.0 (−37.0 to −14.0)	−25.0 (−34.8 to −12.2)	−27.0 (−37.0 to −14.0)
Days b/w treatment start and FU1 [median, IQR]	68.0 (46.0–77.2)	67.0 (46.5–73.0)	68.0 (46.0–78.0)

### Image Registration Performance

We evaluated the performance of the registration algorithm merely to identify the cases where the registration algorithm failed. The evaluation of a registration algorithm is usually performed by evaluating the distance between two known corresponding landmarks in the registered image. This can be done automatically, in a circular fashion. Namely, by selecting N random points in an image, we can transform them to their new coordinates in the target image, and back, using the registration functions *T_AB_*to represent the transformation from source to target, and *T_BA_* as the transformation from target to source. Ideally, these should be the inverse of one another. Practically however there is a registration error propagating from source to target and back. We estimate this error to be proportional to the euclidean distance between *N* and *T_BA_(T_AB_(N))*. It is not exactly the registration error, as this depends on two subsequent dependent registration steps. However, as registration is merely the auxiliary task in our model, a full evaluation of the registration procedure—also in terms of architecture and network components—is beyond the scope of this study. The purpose of this analysis is to analyze the worst cases, i.e., the failures of the algorithm.

We ran the evaluation for all scan pairs, with 100 randomly generated points that were transformed from prior to subsequent, and back to prior. The resulting error was 1.67 cm, on average (CI: 0.87–3.18). We selected for visual inspection the three worst cases, with error 4.54, 3.76 and 3.75 cm, respectively (see figure in **Supplement 2**). These can be considered the closest case of failure of the algorithm. In each of these cases, we can notice the presence of unlikely deformation, like in the heart or the thoracic wall. Although a penalty was set to deter this behavior, we would refrain from increasing it, as it might limit the ability of the network to model other deformations. The strength of the algorithm is represented by the classifier able to distinguish informative deformations from non-informative ones. Overall, in other locations of the image, the registration was still successful in matching anatomical structures properly.

### Prognostic Performance

We fed pairs of subsequent follow-up scans to our network trained for (CT chest) image-to-image registration, and trained a random forest classifier (RFC) on its feature maps to investigate the prognostic value of the imaging features learned by the network. Overall results of the RFC survival score on the independent validation set show an AUC of 0.68 (N = 335, CI: 0.62–0.74, *p <* 0.001) to predict 1-year overall survival from the date of the later scan of the scan pair (see **Figure 3B**). The highest prognostic value can be found for the scan pair BL-FU1, reaching an AUC of 0.74 (N = 61, CI: 0.61–0.86, *p <* 0.001), and for the scan pair FU1-FU2, reaching an AUC of 0.75 (N = 42, CI: 0.58–0.89, *p =* 0.002). A decrease in performance is observed during follow-ups, with a 0.71 AUC (N = 42, CI: 0.50–0.89, *p =* 0.02) for the pair FU2-FU3. None of these differences however reached statistical significance. Interestingly, RFC survival scores on the pair PBL-BL also showed prognostic value (0.69 AUC, N = 51, CI: 0.54–0.83, *p* = 0.01). After the fourth follow-up image, the prognostic performance of the model dropped (0.57 AUC, N = 131, CI: 0.47–0.67, *p =* 0.11). This trend becomes evident when looking at the performance with respect to the days between the later scan in the scan pair, and the start of the treatment (see **Figure 3C**). In this respect, we divided the exam pairs in five groups, based on the time between the day of the later scan, and the day of start of treatment (i.e., before start of treatment, 0–90 days from start, 90–180 days and >365 days), and tested the performance in each group individually. Exam pairs performed before start of treatment showed an AUC of 0.72 (N = 48, CI: 0.57–0.86, *p =* 0.006), between start and 90 days after start of treatment showed an AUC of 0.73 (N = 64, CI: 0.59–0.84, *p <* 0.001), between 90 and 180 days showed an AUC of 0.68 (N = 59, CI: 0.51–0.83, *p =* 0.01), between 180 and 365 days an AUC of 0.66 (N = 89, CI: 0.51–0.79, *p =* 0.01). Exam pairs performed in the second year of treatment showed an AUC of 0.63 (N = 75, CI: 0.50–0.75, *p =* 0.04). Results summary in **Table 2**.

**Table 2:** Performance of the AI model in predicting 1 year survival after the date of the CT scan.

	N -	N +	*p*-value	Area under the ROC curve
All	128	207	<0.001	0.68 (CI: 0.62–0.74)
PBL-BL	27	24	0.010	0.69 (CI: 0.54–0.83)
BL-FU1	30	31	<0.001	0.74 (CI: 0.61–0.86)
FU1-FU2	18	32	0.002	0.75 (CI: 0.58–0.89)
FU2-FU3	14	28	0.015	0.71 (CI: 0.50–0.89)
FU3 +	39	92	0.112	0.57 (CI: 0.47–0.67)
*With respect to days from start of treatment*				
< 0	25	23	0.0057	0.72 (CI: 0.56–0.86)
0–90	33	31	<0.001	0.73 (CI: 0.60–0.84)
90–180	19	40	0.013	0.68 (CI: 0.51–0.83)
180–365	26	63	0.011	0.66 (CI: 0.51–0.79)
365 +	25	50	0.037	0.63 (CI: 0.50–0.75)

### Biomarker Performance

To investigate the prognostic value of AI-monitoring as a biomarker we ran a survival analysis on the scan pairs closest to the date of treatment start, i.e., PBL-BL and BL-FU1. High and low-risk groups were defined for each scan pair by splitting the RFC survival scores on the median value. The scan pair BL-FU1 offered the highest prognostic performance (*p =* 0.02), with a median survival difference of 357 days (637 vs 280 days median survival respectively, *p =* 0.02, see **Figure 3D**). A similar trend was observed for the PBL-BL pair, with a median survival difference of 239 days (467 vs 228 days median survival, respectively, see **Figure 3E**). This, however, did not reach statistical significance (*p =* 0.16). For durable clinical benefit (6 months progression-free survival from start of treatment), we ran a classification analysis on the same scan pairs. This yielded a significant performance of 0.67 AUC (CI: 0.52–0.80, *p =* 0.01) for the BL-FU1 pair, and a similar trend for the PBL-BL pair (0.61 AUC, CI: 0.44–0.77, *p =* 0.10).

### Combination of Multiple Time-points

To investigate the prognostic value of AI-monitoring across multiple time points, we combined the prognostic scores of PBL-BL monitoring, and BL-FU1 monitoring (see **Figures 3F, G** **)**. For this particular analysis, we chose the start of treatment as reference, as differences in follow-up schemas might magnify when combining multiple time-points. Across the subset of patients analyzed (with PBL, BL and FU1 scans available, N = 43), 53% survived 1 year after start of treatment (N = 23). Patients with high expression of prognostic features during the monitoring of both PBL-BL and BL-FU1 (N = 15) showed the highest increase in survival, with enrichment from the baseline of 27% (80% survived 1 year after start of treatment). On the contrary, patients with low prognostic features on both PBL-BL and BL-FU1 (N = 14) showed a diminution from baseline of 24% (29% survived 1-year after start of treatment). A point of interest is to be made for patients showing conflicting prognostic scores between PBL-BL and BL-FU1 (positive-negative and negative-positive, N = 7, respectively). While these groups do not seem to show any deviation from the baseline (50% survived 1-year after start of treatment), further analysis on OS showed comparable results to the negative-negative group (*p =* 0.99) over a longer time span (2 year, see **Figure 3H**). The positive-positive group, on the other hand, kept showing significantly higher OS compared to both negative-negative (*p =* 0.01) and negative-positive (*p =* 0.003) groups.

### Comparison With Known Prognostic Factors

To compare the prognostic value of AI-monitoring against other known clinical prognostic factors, we ran a multivariate cox-hazards survival analysis. Specifically, we compared the RFC prognostic scores to age, cancer subtype, volumetric changes in total tumor burden between BL and FU1, and PDL1 expression at baseline. To mitigate collinearity, we reduced PBL-BL/BL-FU1 scores to a single score by principal component analysis. Complete data was available for 22 patients in the independent validation set. Results showed our RFC survival score preserved statistical significance (0.35 HR, CI: 0.12–0.97, *p =* 0.04) against age (2.69 HR, CI: 1.20–6.05, *p =* 0.02), volumetric change of total tumor burden (2.36 HR, CI: 0.67–8.22, *p =* 0.18), >1% PDL-1 expression (0.26 HR, CI: 0.03–2.22, *p =* 0.22), adenocarcinoma (0.34 HR, CI: 0.03–4.43, *p =* 0.41) and squamous subtype (0.14 HR, CI: 0.01–3.01, *p =* 0.21).

### Visual Inspection of Prognostic Maps

The main idea behind predictive maps was to evaluate the predictive value of different regions of the image by removing those regions, one at a time, and estimating the difference in predicted survival. **Figure 3I** shows an example. The input scans are displayed in the first column. The second column shows the prognostic map generated by the occlusion algorithm (**Supplement 1**). The patchy look of the overlay is the result of the cubic ROI, being scrolled around the image. Its intensity values are proportional to the change in predicted survival resulting from occluding that region. The third column is the deformation map, where hotspots correspond to regions of gross morphological changes (i.e., pleural effusion). The fourth column was the visualization presented to the reader. It is the result of the fusion between the prognostic map and the deformation map, and highlights the prognostic changes identified by the algorithm.

At visual inspection, lymph node metastases and lung lesions were common hotspots in the prognostic maps. Nodal metastases were present in 58% of scan pairs (N = 57), and highlighted as prognostic in 81% of the cases (N = 46). The mediastinum contained the most nodal hotspots, being highlighted in 80% of cases, followed by supraclavicular and hilar nodal metastases, highlighted in 67% and 57% of cases respectively. Axillary and pericardial nodal metastases were hotspots in 75% and 50% of cases, but found only in 4 and 2 scan pairs respectively. Large lung masses were found in 45% of scan pairs (N = 39), and highlighted as prognostic in 85% of cases. The same rate was observed for small lung nodules, while being less frequent, found in 30% of the scan pairs (N = 26). Bone metastases were found in 20% of scan pairs (N = 17). Nonetheless, they were deemed prognostic by the algorithm in 82% of cases. Pleural masses, liver metastases and subcutaneous lesions, while being almost exclusively hotspots in the prognostic maps, accounted together for only 13 scan pairs. Among secondary comorbidities, pleural effusion, consolidations and atelectasis were the most common, accounting for 31%, 28% and 20% of scans pairs (N = 27, 24, and 17, respectively). Hotspots were found in 94% cases of atelectasis (N = 16), 93% cases of pleural effusions (N = 25), and 83% cases of non-specific consolidation (N = 20). Pericardial effusions were hotspots in 75% of the times, but found only in 8 cases. Only one case of ascites was reported, which the algorithm also highlighted as prognostic. Hotspots in anatomical regions included the spine in 56% of cases, the thoracic wall in 55% of cases, and various regions in the upper thorax, including periscapular (51%), shoulders (49%), neck (48%), and supraclavicular (45%), with the exception of the axilla, highlighted only in 13% of scan pairs. Normal lung parenchyma was highlighted in 28% of cases. The remaining hotspots include the great vessels (9%) and the breast (4%). Detailed summary reported in **Table 3**.

**Table 3:** Highlighted areas in the AI-generated prognostic maps.

	ALL	PBL-BL	BL-FU1
***Tumor Related***			
Lymph Nodes	46/57 (80.70%)	21/27 (77.78%)	25/30 (83.33%)
— Pericardial	1/2 (50.00%)	1/1 (100.00%)	0/1 (0.00%)
— Mediastinal	42/53 (79.25%)	18/25 (72.00%)	24/28 (85.71%)
— Hilar	16/28 (57.14%)	7/12 (58.33%)	9/16 (56.25%)
— Supraclavicular	16/24 (66.67%)	5/10 (50.00%)	11/14 (78.57%)
— Axillary	3/4 (75.00%)	1/2 (50.00%)	2/2 (100.00%)
Large Lung Masses	33/39 (84.62%)	16/20 (80.00%)	17/19 (89.47%)
Small Lung Nodules	22/26 (84.62%)	8/11 (72.73%)	14/15 (93.33%)
Bone Metastases	14/17 (82.35%)	7/7 (100.00%)	7/10 (70.00%)
Pleural Masses	6/6 (100.00%)	3/3 (100.00%)	3/3 (100.00%)
Liver Metastases	5/6 (83.33%)	2/3 (66.67%)	3/3 (100.00%)
Subcutaneous Lesions	1/1 (100.00%)	—	1/1 (100.00%)
***Secondary Comorbidities***			
Pleural Effusion	25/27 (92.59%)	12/12 (100.00%)	13/15 (86.67%)
Consolidation	20/24 (83.33%)	10/12 (83.33%)	10/12 (83.33%)
— Post-radiation	3/3 (100.00%)	2/2 (100.00%)	1/1 (100.00%)
Atelectasis	16/17 (94.12%)	9/9 (100.00%)	7/8 (87.50%)
— Post-obstructive	7/8 (87.50%)	4/4 (100.00%)	3/4 (75.00%)
Pericardial Effusion	6/8 (75.00%)	2/3 (66.67%)	4/5 (80.00%)
Ascites	1/1 (100.00%)	—	1/1 (100.00%)
***General Anatomical Areas***			
Spine	48/86 (55.81%)	26/43 (60.47%)	22/43 (51.16%)
Thoracic Wall	47/86 (54.65%)	25/43 (58.14%)	22/43 (51.16%)
Periscapular	44/86 (51.16%)	20/43 (46.51%)	24/43 (55.81%)
Shoulder	42/86 (48.84%)	23/43 (53.49%)	19/43 (44.19%)
Neck	41/86 (47.67%)	20/43 (46.51%)	21/43 (48.84%)
Periclavicular	39/86 (45.35%)	19/43 (44.19%)	20/43 (46.51%)
Lung Parenchyma	24/86 (27.91%)	13/43 (30.23%)	11/43 (25.58%)
Axilla	11/86 (12.79%)	6/43 (13.95%)	5/43 (11.63%)
Great Vessels	8/86 (9.30%)	5/43 (11.63%)	3/43 (6.98%)
Breast	3/86 (3.49%)	1/43 (2.33%)	2/43 (4.65%)

## Discussion

Advanced treatment monitoring through more detailed quantitative descriptors of the overall status of the patient, as visualized on routine imaging scans, could provide valuable prognostic information. Our aim was to investigate the potential prognostic value gained by AI-based treatment monitoring on imaging in NSCLC patients treated by PD-1 checkpoint inhibitors. To test this, we implemented a convolutional neural network for image-to-image registration, and trained it on a large public dataset of chest CT scans. The trained network was then used to longitudinally model gross morphological changes between subsequent scans of NSCLC patients receiving PD1 checkpoint inhibitors. Morphological changes identified by the network were then used to train a classifier to predict 1-year OS from the date of the latest scan.

Our results showed significant performance in the independent test set for the prediction of 1-year OS from the date of image acquisition, with an average AUC of 0.69, and up to 0.75 AUC for the first 3 to 5 months after start of treatment, and 0.67 AUC for durable clinical benefits, suggesting the presence of (AI-quantified) gross morphological changes encoding prognostic value. These results are comparable to state-of-the-art methods, which currently employs laborious and time-consuming segmentation procedures (11, 12). While the field of research has been focusing on single-lesion analysis—leveraging different known factors in cancer growth, including vascularity (30), oxygenation (31), and metabolic activity (32)—our approach offers a novel fully automatic procedure which completely eradicates the need of time-consuming segmentations, and simultaneously offers a way to provide a full picture of the patient status as seen on chest imaging. While this does not preclude the usefulness of the single-lesion approach, it proposes a way for future multi-scale solutions that leverage both single lesion imaging biomarkers as well as whole image approaches that provide general quantitative information about the status of the patient receiving treatment. Research efforts, however, have to be made in order to overcome the bottleneck of manual ROI delineation procedures, either in the form of automatic segmenters (33), or with implicit AI representations of the total tumor burden.

In addition to the statistical analysis of the performance, we investigated the choices the AI made by means of sensitivity occlusion (29). This resulted in a set of prognostic heatmaps, highlighting regions of morphological changes that the AI deemed prognostic relevance. Gross morphological changes in nodal and lung lesions held the highest prognostic value, especially nodal lesions in the mediastinum, hilum, and supraclavicular region. Further results suggested additional prognostic value for morphological changes affecting the lungs, either in the form of compression from the thoracic wall (due to pleural effusion or pleural masses), non-specific consolidations, or atelectasis. These results also seemed to extend to other regions, with ascites and pericardial effusions also being highlighted as prognostic, despite their rare occurrence. The AI seemed to pay particular attention to the skeleton, with the spine being the anatomical region most commonly highlighted by the AI in the prognostic maps, and bone metastases deemed prognostic in most cases where those were present. As common imaging follow-up schemas, such as RECIST (29, 34), do not account for tumor burden in the bones, our findings suggest that, on the contrary, such phenomena should not be ignored. Further investigations should lead to novel guidelines, which can provide valuable contribution from the imaging beyond diametrical measurements.

Particular attention should also be paid to nodal metastases and nodal growths during treatment. Imaging features of nodal metastases were found already to be correlated with disease progression for NSCLC, melanoma, and head and neck cancer (11, 35), though no distinction was made between the location of the lymph nodes. However, both our findings and the current literature suggest that this information may be of value. This would be especially interesting in the light of regional (tumor-draining) lymph nodes which play a critical role in terms of anti-tumor immunity and priming (36), increased expression of cytokines and checkpoint markers (37), and changes in the immune compartments resulting in a tumor favorable microenvironment (38). A major hurdle that remains in the analysis of lymph nodes is represented by the radiological assessment, often in contrast with the pathological one. Most radiomics studies so far focused on the detection of positive nodal metastases rather than their prognostic values (39–44).

The analysis of lung lesions is far more common. Imaging features from lung lesions have been reported to hold prognostic value for patients receiving immunotherapy in several studies (11, 32, 45–48). Indeed our findings confirm the association between lung lesions and treatment outcome, with about 85% percent of them being hotspots in the AI-generated prognostic maps, independent of size. Most of the studies published so far focus on the analysis of the tumor region and/or the peritumoral boundary, which may hold valuable information regarding tumor vascularization and inflammatory environment. In this study, the proposed AI model monitors the whole image including both the healthy tissue as well as the tumor(s). As the growth of a cancer lesion does not uniquely depend on the genetic makeup, but rather a complex interaction of microenvironmental features and favorable location for seeding, it would not be surprising to establish a link between a comprehensive modeling tool of cancer growth and its biological features. Even in this case however, further research is needed to establish any link between imaging features and tumor biology.

Following our results, we observed an increase in the prognostic performance of the AI resulting from the combination of multiple time points, namely pre-baseline, baseline and first follow-up. This analysis showed good OS for patients with higher AI-survival scores (AI-RFC^hi^) in both pre-baseline to baseline scan pair, and baseline to first follow-up—and worse OS for the opposite case (AI-RFC^lo^). Interestingly, patients with contradicting scores (AI-RFC^hi^ for pre-baseline to baseline scan pair, and AI-RFC^lo^ for the baseline to follow-up, and vice versa) showed worse survival, similar to the double negative group. These results suggest the existence of a prognostic combination of pretreatment and early-treatment characteristics, both of which should be accounted for during patient stratification. Further insights could be achieved by more advanced AI methods that would account for larger time spans, or even the entirety of patients’ treatment history.

The combined score was demonstrated to be an independent prognostic parameter even when corrected for other known prognostic parameters. This is of particular interest when we consider the possible role of such a tool, for example as an additional input to the tumor board during treatment decision making. Further research is required to study its implementation in the clinical settings.

### Limitations and Future Outlook

Our study aimed to monitor AI-measured gross morphological changes between imaging follow-up for survival prediction in NSCLC patients receiving PD1 checkpoint antibodies. In this study, we pre-trained a neural network on a large dataset of chest CT scans, and fine-tuned it for survival on our smaller local immunotherapy data set. Under the current settings, we limited the analysis to chest imaging which, in addition to the chest, frequently included the lower neck and the upper abdomen. While this limitation could hold for lung malignancy, an extension to other cancer types would require this technique to be extended to include the whole body—i.e., the abdomen and, when available, the brain. Moreover, due to the limited amount of data, it was not possible to explore more complex machine learning algorithms for the prediction of survival, nor for more precise visualization of the prognostic maps. Expansion of the dataset, both in terms of patients and in terms of time points, would certainly allow for an increase in performance and better explainability of the AI algorithm. Specifically, an extension of the field of view of the algorithm to the whole body, as well as the usage of parameters other than imaging, could potentially improve the performance of the algorithm to be usable in the clinics. Further clinical validation of the method is also needed. While this study presented a comparison of this method with response evaluation criteria (e.g., changes in total tumor burden) and biomarkers (e.g., PD-L1 expression), the primary objective for future studies should be a comparison with the clinical standard, namely the RECIST criteria. It remains to be investigated whether this method would be complementary to the current radiological response evaluation (i.e., RECIST). Furthermore, additional investigations are required to link biological features to tumor growth and gross morphological changes. Further analysis should also study the effects of different machine acquisition parameters, and the sensitivity of the method to imaging acquisition parameter variability. Looking into the future, we envision that an AI solution could be set up as a clinical decision support system capable of providing information to the treating physician complementary to traditional clinical and pathological input data.

## Conclusions

In this study, we aimed to investigate the potential prognostic value of AI-mediated monitoring in NSCLC patients receiving PD-1 blockade. We hypothesized the existence of quantitative imaging features describing a set of gross morphological changes happening during treatment that hold prognostic information. Our results demonstrate the existence of such factors (as described by the AI on imaging), that are tumor-related, such as nodal, lung and bone lesions, as well as non-tumor related, such as pleural effusions, atelectasis and non-specific consolidations. Further investigation should focus on the development of more flexible models that can extend beyond thoracic imaging, as well as on external validations.

## Data Availability Statement

The data sets presented in this article are not readily available because the restrictions of agreement with the local ethical committee. We can, however, share the feature space on request. 

## Ethics Statement

The studies involving human participants were reviewed and approved by Institutional Review Board. Written informed consent for participation was not required for this study in accordance with the national legislation and the institutional requirements.

## Author Contributions

ST: software development. ST, ZB, TT, TDLN-K: conceptualization, experimental design. ZB, TB, TDLN-K: clinical results validation and interpretation. ST, ZB, SD, IK, AC-V, AD, TDLN-K: radiological and clinical data collection and curation. MM, KH: pathological and clinical data collection and curation. KJH, TDLN-K, ES, HA, RB-T: project supervision, resource acquisition. All authors: results and manuscript editing and validation. All authors contributed to the article and approved the submitted version.

## Funding

This work was also carried out on the Dutch national e-infrastructure with the support of SURF Cooperative. The authors would also like to thank NVIDIA for their kind donation *via* the Academic GPU Grant Program as well as the Maurits en Anna de Kock Stichting for its financial support. TN-K was funded by the Oncologic Imaging Fellowship Grant from the European Society of Radiology.

## Conflict of Interest

Author HJWLA is an advisor for Onc.Ai and Bristol Myers Squibb (BMS), outside of submitted work. Author AD was employed by company Affidea.

The remaining authors declare that the research was conducted in the absence of any commercial or financial relationships that could be construed as a potential conflict of interest.

## Footnotes

1 Github: github.com/nki-radiology/PAM
