# Supplementary material for: Prognostic Value of Deep Learning-Mediated Treatment Monitoring in Lung Cancer Patients Receiving Immunotherapy
Source: Front Oncol. 2021 Mar 2;11:609054. doi: 10.3389/fonc.2021.609054 (PMC7962549; doi:10.3389/fonc.2021.609054)
Supplement: Supplementary file 1 [file DataSheet_1.pdf]

S1. Generation of Heatmaps for Model Explainability

***Input** (prior, subsequent, time_interval, time_start)*

1 **Reference score** → survival score on the original images

2 **ROI** → cube of 64x64x64 in the top left back corner

3 **Occluded images** → set intensities within the **ROI** to zero in both prior and subsequent scan

4 **Occluded score** → Compute the survival score on the occluded_images

5 **ROI importance** → | occluded_score - reference_score |

6 **Prognostic map[ROI]** → maximum(prognostic_map[ROI], roi_importance) [A]

7 Move the **ROI** 8 voxels along one of the axis

8 If **ROI** has not scrolled through the whole image yet, go to **Step 3**

9 **Deformation map** → anatomical changes between prior and subsequent, as returned by the registration

10 **Return** guassian_filter(deformation_map X prognostic_map) [B]

[A] Since the ROI are overlapping, we chose to use the maximum function, which prevents erroneous overriding of previous estimation. [B] We fuse the prognostic map and the deformation map together (S3-4.0) to refine the hotspots to regions of changes, and therefore help along the radiologist in the visual interpretation.
