# Supplementary material for: Prognostic Value of Deep Learning-Mediated Treatment Monitoring in Lung Cancer Patients Receiving Immunotherapy
Source: Front Oncol. 2021 Mar 2;11:609054. doi: 10.3389/fonc.2021.609054 (PMC7962549; doi:10.3389/fonc.2021.609054)

S2. Illustration of the 3 worst case of image registration performance

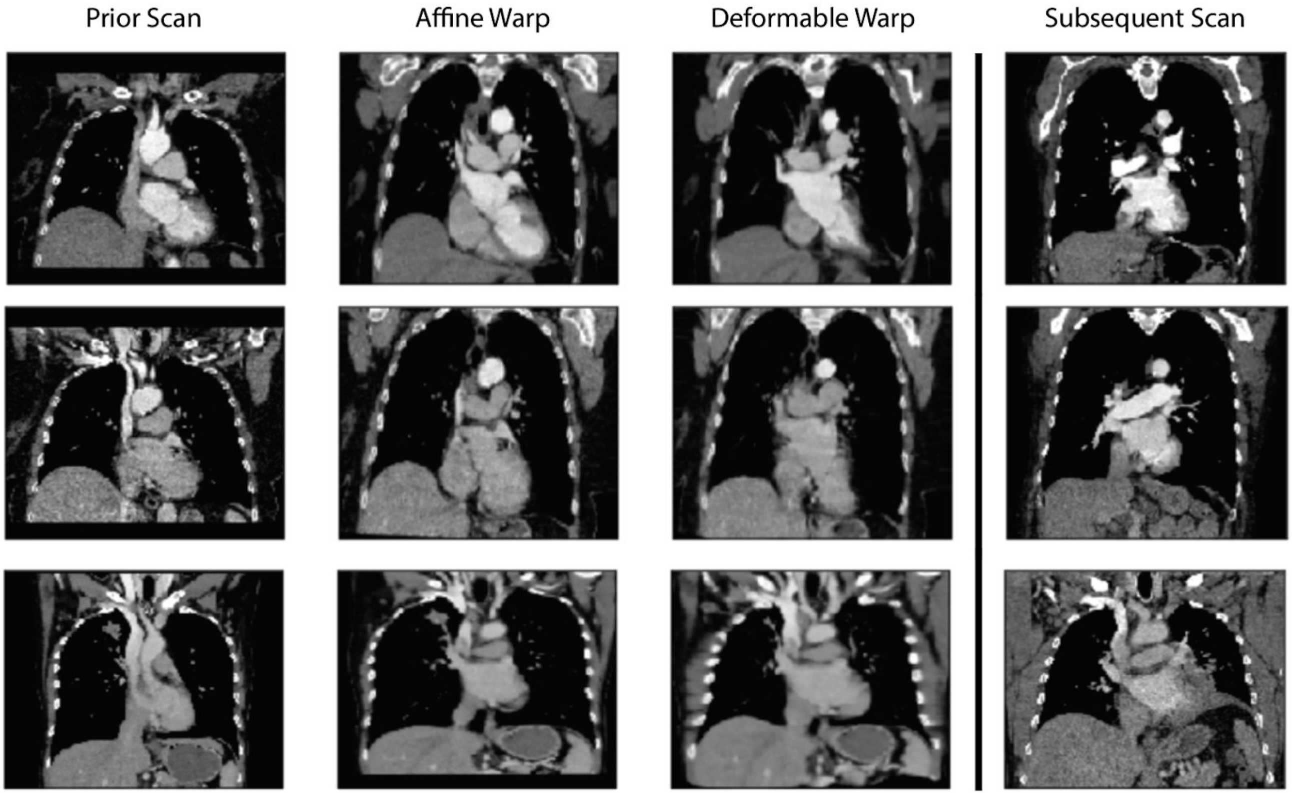

Supplement: Supplementary file 2 [file DataSheet_2.pdf]
